# Readmission rates following heart failure: a scoping review of sex and gender based considerations

**DOI:** 10.1186/s12872-020-01422-3

**Published:** 2020-05-14

**Authors:** Amy Hoang-Kim, Camilla Parpia, Cassandra Freitas, Peter C. Austin, Heather J. Ross, Harindra C. Wijeysundera, Karen Tu, Susanna Mak, Michael E. Farkouh, Louise Y. Sun, Michael J. Schull, Robin Mason, Douglas S. Lee, Paula A. Rochon

**Affiliations:** 1 Women’s College Research Institute, Toronto, Canada; 2 Peter Munk Cardiac Centre of University Health Network, Toronto, Canada; 3 ICES, Toronto, Canada; 4 Institute for Health Policy, Management and Evaluation, University of Toronto, Toronto, Canada; 5 Faculty of Medicine, University of Toronto, Toronto, Canada; 6 Ted Rogers Centre for Heart Research, Toronto, Canada; 7 Sunnybrook Health Sciences Centre, Toronto, Canada; 8 North York General Hospital, Department of Family and Community Medicine, University of Toronto, Toronto, Canada; 9 Sinai Health System, Toronto, Canada; 10 Division of Cardiac Anesthesiology, University of Ottawa Heart Institute, Ottawa, Canada

## Abstract

**Background:**

Although hospital readmission for heart failure (HF) is an issue for both men and women, little is known about differences in readmission rates by sex. Consequently, strategies to optimize readmission reduction programs and care strategies for women and men remain unclear. Our study aims were: (1) to identify studies examining readmission rates according to sex, and (2) to provide a qualitative overview of possible considerations for the impact of sex or gender.

**Methods:**

We conducted a scoping review using the Arksey and O’Malley framework to include full text articles published between 2002 and 2017 drawn from multiple databases (MEDLINE, EMBASE), grey literature (i.e. National Technical information, Duck Duck Go), and expert consultation. Eligible articles included an index heart failure episode, readmission rates, and sex/gender-based analysis.

**Results:**

The search generated 5887 articles, of which 746 underwent full abstract text consideration for eligibility. Of 164 eligible articles, 34 studies addressed the primary outcome, 103 studies considered sex differences as a secondary outcome and 25 studies stratified data for sex. Good inter-rater agreement was reached: 83% title/abstract; 88% full text; kappa: 0.69 (95%CI: 0.53–0.85). Twelve of 34 studies reported higher heart failure readmission rates for men and six studies reported higher heart failure readmission rates for women. Using non composite endpoints, five studies reported higher HF readmission rates for men compared to three studies reporting higher HF readmission rates for women. Overall, there was heterogeneity between studies when examined by sex, but one observation emerged that was related to the timing of readmissions. Readmission rates for men were higher when follow-up duration was longer than 1 year. Women were more likely to experience higher readmission rates than men when time to event was less than 1 year.

**Conclusions:**

Future studies should consider different time horizons in their designs and avoid the use of composite measures, such as readmission rates combined with mortality, which are highly skewed by sex. Co-interventions and targeted post-discharge approaches with attention to sex would be of benefit to the HF patient population.

## Brief summary

There has been increased attention on reducing hospital readmission rates. In this scoping review, we found notable variation exists in studies with sex stratified analyses for HF patients readmitted to hospital. Few studies were prospective in design, and results could be conflated by reporting of composite measures and time to event. Co-interventions and targeted post-discharge approaches need to be revisited for men and women.

## Background

The rise in hospital readmissions is a global concern, placing considerable burden on patients, treatment costs, and hospital resources [1]. In the United States, the 30-day readmission rate for those with heart failure (HF) increased from 17 to 20% between the years 1993 and 2006 [2]. The number of people living with HF is increasing, and age/sex-standardized prevalence of the condition has been relatively stable over time [3]. Current patterns of hospital readmission are often associated with organizational factors, such as a length of stay, and clinical factors, such as age and comorbidities [4]. Improved quality of care at patient intake is also associated with a lower probability of readmission [5, 6]. Strategies to reduce readmission rates have shifted from hospital-based to more patient-centred strategies, such as telemonitoring, which may benefit patients by facilitating their access to health care services [7, 8]. Public reporting and financial incentives have been trialed by governments with the intent to reduce hospitalization rates [9, 10].

Heart failure is a growing problem with similar prevalence in men and women [11–13]. However, information on processes, quality of care, health status outcomes, or other patient care experiences has not been explored in the context of sex and gender. Heart failure with preserved ejection fraction (HFpEF) is more prevalent in women [13]. The lifetime risk of heart failure is 15% for women and 11% for men for those without a history of myocardial infarction at age 40 [8]. A few authors have focused on sex differences in heart failure [14–17] but no study to date has examined this in relation to readmission rates. This article draws on a scoping review protocol to better understand current patterns of readmission and the interpretation of observed patterns in relation to sex. Our aim was to examine studies reporting a higher heart failure readmission rate for either women or men, and to provide a qualitative overview of the possible considerations for the impact of sex and gender on this outcome.

## Methods

### Study protocol

A scoping review protocol was developed using the methodological framework proposed by Arksey and O’Malley [18]. In addition to the aforementioned methods, we also used the Joanna Briggs Institute Methodology for scoping reviews [19] and further refined the process using recommendations put forth by Levac et al. [20] This scoping review is related to the COACH trial (clinicaltrials.gov NCT02674438- Last accessed October 2, 2017). A consultation of experts was included to inform the search for additional articles of interest.

### Information sources and literature search

The search strategy was developed in consultation with the research team and was peer-reviewed by an expert librarian using the PRESS peer-review of electronic search strategy checklist [21] (Appendix). Multiple databases were searched, from 2002 up to May 29, 2017, including MEDLINE, EMBASE, PubMed, CINAHL, and Web of Science. Experts were then consulted up until October 16, 2017 for additional articles of interest (Table 1). Keywords and combinations of mesh terms were used to narrow the search strategy: “heart failure” OR “systolic dysfunction” OR “diastolic dysfunction” OR “heart ventricular failure” OR “left ventricular dysfunction” OR “cardiac failure” OR “cardiac decompensation” OR “heart decompensation” was combined with Readmission* or readmit* or rehospital* and sex, gender or male/female. Additional filters narrowed the results to those papers published in the past 15 years and limited to humans. The database search was supplemented by a manual search of related references in the literature drawn from the Sex and Gender-Specific Medicine (SGSM) PubMed database (Texas Tech University).

**Table 1:** Study characteristics

Study Characteristics	Type, n
Journal disciplines	Peer-reviewed or double blind, *n* = 34 Dissertations, *n* = 2
Grey Literature (n = included/potentially eligible)	CADTH- Canadian Agency for Drugs and Technologies in Health *n* = 0/15
	Center for Disease Control n.r^a^.
	CIHI n.r.
	Health Canada n = 0/9
	Google Scholar *n* = 1/491
	TRIP n = 1/19
	National Technical Information Service n = 1/581
	Scopus n = 1/10
	Duck Duck Go n = 2/200
	UofT Theses and Dissertations in the Sciences n = 0
	OAIster n = 0/2
	Health Quality Ontario, n = 0/5
	New York Academy of Medicine’s Grey Literature Report n = 0
	Open Grey n = 0/4
	T-space n = 0/31
	World Health Organization n.r.
Consultation Experts	Journal Articles 1/7 (exclusion criteria: did not address re-admission)
Study Design	Prospective *n* = 11
	Retrospective *n* = 25
Year of publication	2013–2017 *n* = 14
	2008–2012 *n* = 13
	2002–2007 *n* = 9
Geographical region	North America (US n = 14, Canada *n* = 4)
	Europe n = 11
	Asia n = 4
	Australia n = 2
	Africa n = 1

^a^*N.r.* Not reported

### Eligibility criteria

We included reports, both published and unpublished, that were primary research and in English. Further consideration for inclusion was whether the results disaggregated for sex or gender in readmission. Articles were excluded if they did not include HF as a first episode, enrolled exclusively a pediatric population (or those less than 18 years of age), was not full text, or published more than 15 years prior to the time the search was performed.

### Study selection process

The screening criteria were established a priori and calibrated amongst the team (DSL, RM, PR, AHK, CP) with a pilot-test by screening title and abstract on a random sample of 220 articles and 100 full text articles. After an acceptable inter-rater agreement was established at 80%, pairs of reviewers (CP, AHK) screened the pool of potentially eligible articles.

### Data items and data abstraction process

We employed a ‘descriptive-analytical’ method, a narrative study design, which applies a common framework to all the included studies [20]. A sample of articles was read, and a data abstraction chart was developed. Charting is a technique for synthesizing and interpreting qualitative data by sifting and sorting materials by key issues and themes [19, 22]. We charted data into categories of study design, objectives (primary, other), study population, setting, sample estimation, endpoints, and statistical methods for sex group differences. Charting was stratified by sex for sample size, age, sample lost to follow up, event-free survival, mortality rate, all-cause and heart failure readmission rates, non-cardiovascular readmission rates, and any other potentially important findings. Length of hospital stay, time to event, and hazard ratios for readmission were also extracted. The chart was calibrated by the team (WW, RM, PR, DL, AHK, CP) on a sample of 12 included articles (11 from the sex and gender-specific medicine PubMed database and 1 article from the grey literature). Two reviewers used a standard form to decide which resources contained a primary objective evaluating sex differences in patients with heart failure following readmission.

**Fig. 1:**
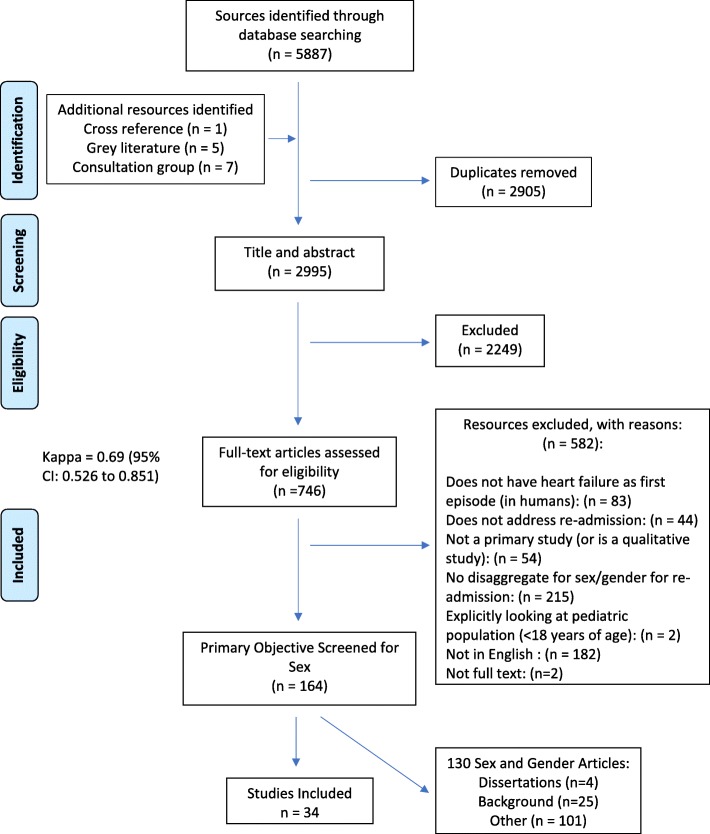
Prisma flow diagram showing the process used for screening studies using sex-gender analysis for HF readmissions

When more than one outcome was reported in an article, we used a hierarchical selection process: (a) authors’ explicit declaration of primary objective, (b) the outcome used to calculate the sample size, (c) authors’ attribution of importance to the outcome in their description of the results, or (d) the outcome that appeared first in the methods section [23]. We used the category “Other” to classify resources that looked at patient populations presenting with a first episode of heart failure, if sex differences following readmission was specified as a secondary outcome measure or other (Fig. 1). Since studies with composite outcomes can mask associations that might be directionally opposite for death versus readmission, we determined whether composite outcomes were used in combination with mortality. We used the category “Background” to classify resources that contained information about readmission rates for men and women, but the authors did not perform sex-stratified analyses. Disagreements were resolved by discussion and other authors were involved to assist with ambiguous cases.

### Definition of sex and gender

Although the number of publications that are relevant to sex/gender in the field of HF has grown over the years (Table 1), we found that there is still much confusion over terminology. For consistency, in this review “sex” will refer to a set of biological attributes in human participants that are associated with physical and physiological features [24]. The term “gender” will refer to socially constructed roles, relationships, behaviours, and powers as defined by the Government of Canada [24]. However, the data charted are reported per verbatim as used by the authors of the full text resources.

## Results

### Article types

The electronic database search generated 5887 resources. Six additional potential records were identified by a hand search of references from the included articles, the grey literature, and by consulting a group of experts in the field. After duplicates were removed and resources were screened for eligibility, 36 sources remained, comprising 34 journal articles and 2 dissertations (Fig. 1). Good inter-rater agreement for a pair of reviewers was reached at each stage of the eligibility assessment (83% title/abstract; 88% full text; kappa = 0.69 [95% CI: 0.53–0.85]). Most of the sources were recently published: 14 (2013–2017), 13 (2008–2012) and 9 (2002–2007) (Table 1). Twenty-five of the studies were retrospective and 11 were prospective in study design. Most of the studies were from North America including four from Canada. One hundred three sources were classified as “Other” and 25 sources were considered “Background”.

### Clinical aims

A broad range of research questions was addressed. Twenty-one study objectives (34%) focused on predictors, risk or prognostic factors, 15 (24%) presented a query into the clinical profiles or etiology of patients readmitted with an index diagnosis of heart failure. Sex differences were explicitly stated only in 12 (19% study objectives). Environmental or external factors such as timing or setting were the least frequently addressed [objectives: 1 (2%) and 2 (3%)], respectively.

### Definition of heart failure

Notably for defining heart failure, none of the included studies considered sex differences in HF presentation or sex-specific characteristics. A large proportion of studies in this scoping review defined HF using the World Health Organization’s International Classification of Diseases code: 14 (35%) studies. The remaining 6 (16%) studies used the Framingham study clinical criteria, 4 (11%) studies, adopted the criteria defined by the European Society of Cardiology, 7 (18%) papers defined HF with clinical examinations, 3 (7%) studies, used specific regional hospital admission codes or registry database codes, in 1 (2%) study, HF was defined as impaired cardiac output, and in 3 (7%) studies, HF was not defined. In addition, 4 (10%) studies reported using multiple diagnostic criteria. HF definitions are summarized in Table 2.

**Table 2:** The primary author and year of publication, and heart failure as defined in the study

Primary Author (Year of Publication)	Definition of heart failure
Macdonald (2008) [25]; Arora (2017) [26]; Omersa (2016) [27]; Howlett (2009) [28]; Madigan (2012) [29]; Robertson (2012) [30]; Blackledge (2003) [31]; Bradford (2016) [32]; Eastwood (2014) [33]; Sheppard (2005) [34]; Chun (2012) [35]; Howlett (2009) [28]; Jenghua (2011) [36]; Lee (2004) [37]	World Health Organization’s International Classification of Diseases code-10th revision; 9th revision; Australian modification
Jimenez-Navarro (2010) [38]; Goncalves (2008) [39]; Ogah (2014) [40]; Opasich (2004) [41]	European Society of Cardiology
Howie-Esquivel (2007) [42]; Sato (2015) [43]; Ogah (2014) [40]; Ogah (2015) [44]; Sajeev (2017) [45]; Lee (2004) [37]	Confirmed by a cardiologist, using standard Framingham criteria or exacerbation of a previously documented HF; Framingham criteria (including symptoms, physical examination, chest x-ray and echocardiographic findings)
Vader (2016) [46]	The presence of more than one symptom (dyspnea, orthopnea, edema) and 1 sign (rales on auscultation, peripheral edema, ascites, or pulmonary vascular congestion on chest radiography) in DOSE-AHF or ROSE-HF or admission to the hospital with a primary diagnosis in CARRESS HF
Gevaert (2014) [47]	Preserved left ventricular fraction was defined as left ventricular ejection fraction greater than or equal to 50% and heart failure with reduced ejection fraction was defined as less than 50%
Chang (2014) [48]	New onset of HF with acute decompensation or chronic HF with acute decompensation requiring hospitalization
Alla (2007) [49]	Current or past evidence of low cardiac output or congestion edema, elevated jugular venous pressure, or rales or evidence of pulmonary congestion
Ieva (2015) [50]	Diagnostic Category - Nervous system, respiratory system, circulatory system, with HF-related events, from Agency for healthcare research and quality and centres for Medicare and medicaid services hierarchical condition category in hospital administrative database
Tarantini (2002) [51]	Hospital admission for CHF
Zsilinskza (2016) [52]	Heart Failure National Registry Emergency Module (ADHERE-EM) database
Sajeev (2017) [45]	Chronic systolic HF was defined as systolic HF for at least 6 months with an EF of less than 50% and/or patients who are on standard HF medications, which include at least two groups of medications mentioned: ACEI/ARB, diuretics or digoxin
Nieminen (2008) [53]	Diastolic dysfunction was classified by the investigator as mild, moderate or severe according to echocardiographic criteria; signs of heart failure (rales, hypotension, hypoperfusion) and signs of heart failure on chest x-ray. Acute decompensated chronic heart failure was defined as worsening of heart failure in patients with a previous diagnosis or hospitalization for heart failure or as new-onset acute heart failure for patients with no prior history of heart failure
Mullens (2008) [54]	Impaired cardiac output (cardiac index < 2.4Lmin/m^2^)
Schwarz (2003) [55]; Otero-Ravina (2009) [56]; Ahmed (2014) [57]	Not reported; Diagnosis of HF made by a specialist (cardiologist and/or internist)

N.B. Does not include information from abstracts only or dissertations, unless linked to published article

### Outcomes reported

Some studies reported readmission outcomes separately while others examined the composite of death or readmission to hospital. Six studies reported that women had higher rates of readmission or experienced the composite outcome more frequently than men (Table 3). Among these 6 studies, 4 had a mean follow-up duration of 3 months or less. Amongst studies in which non-composite outcomes were used, we found 3 that reported higher heart failure readmission rates in women when compared to men [25, 26, 38] (Table 4). One study reported cardiovascular event-free survival (as defined by survival free from HF admission, acute myocardial infarction, coronary revascularization, valvular surgery, or heart transplantation) and found significantly lower risk in women [38].

**Table 3:** Objectives, study population and setting, definition of heart failure as reported in studies reporting higher re-admission rates or composite outcome in women. Studies are listed in alphabetical order by primary author

Primary Author (year)	Objectives	Study Population (interventions)	Study Setting (geographical location, recruitment period)	Study Design	Study Endpoints
Arora (2017) [26]	To evaluate specific etiologies, trends and predictors of 30-day readmission in patients admitted with HF from one of the largest nationwide databases	Patients with heart failure. Besides Medicare, also included Medicaid, private/health maintenance organization and self-pay patients.	2013; all-payer inpatient database in US	Retrospective cohort design	30-day readmissions; with and without HF
Gevaert (2014) [47]	To compare the incidence and treatment of atrial fibrillation on admission between men and women admitted with acute heart failure	Patients included in the prospective BIO-HF registry (evaluate all patients admitted with the New York Heart Association class 3–4)	2 Belgian hospitals, Nov 2006 to May 2012; Patients included in the prospective BIO-HF registry (evaluates all patients admitted with the New York Heart Association class 3–4)	Prospective design	One-year all-cause mortality or readmission for HF. Secondary endpoints were in-hospital mortality and restoration of sinus rhythm at discharge
Howie-Esquivel (2007) [42]	To determine whether demographic, clinical, or psychological variables conferred increased risk of rehospitalization in a multiethnic, hospitalized heart failure population 90 days after hospitalization for heart failure	Patients with HF, English or Korean	Large academic medical center in Northern California, data collected from July 2004 to April 2005	Prospective cohort study	Quality of life, mean discharge brain natriuretic peptide; 6MWT distance, rehospitalizations
Jimenez-Navarro (2010) [38]	To determine the influence of gender on the diagnostic and therapeutic management and long-term prognosis of patients with heart failure seen in specific heart failure clinics	Patients with chronic heart failure. 21% patients were from community hospitals and 79% from the general hospitals.	62 Centers incl. 14 (22%) community hospitals and 48 (78%) general hospitals; 10 (16%) of the participating hospitals have a heart transplantation program. 8 (13% of the total) depend on an internal medicine service. Heart failure units or clinics (Spain, 2000 to 2003)	Retrospective observational multicenter study	Mortality, admissions for heart failure, acute myocardial infarction, coronary revascularization, valvular surgery, or heart transplant
Macdonald (2008) [25]	To assess the association of diabetes with short and long-term outcomes in all patients hospitalized for the first time with heart failure in Scotland	Individuals discharged from hospital with a diagnosis or heart failure according to history of diabetes and sex	Hospitals (Scotland, 1986 to 2003)	Retrospective cohort study	Combined end point of death or HF readmission, also separately reported per males and females
Vader (2016) [46]	Characterized the risk factors for post discharge readmission/death in subjects treated for acute heart failure	Patients hospitalized with acute heart failure	From 3 different trials	Post hoc retrospective analysis	Rehospitalization or death after discharge from the index hospitalization analyzed in a continuous fashion or in the intervals of 0–30 days or 31–60 days

**Table 4:** Studies showing significantly higher rates of outcomes in women, presented alphabetically by primary author

Primary author (year)	Sample Size n (%)	Mean Age (years)	Mean Length of Hospital Stay (days)	Type of Reporting by Sex^**1**^	Time to Event (months), Heart Failure Readmission Rate		Other	Significance
					Female	Male		
Arora (2017) [26]	301,892 F: n (49.4) M: n (50.6)	73.4% ≥ 65 years	5.29 ± 0.01	b, c	1-month, 0.93 OR (0.90–0.96)		Female readmission without HF: 1.02 OR (1.00–1.05)	Females
Gevaert (2014) [47]	957; F:435(44.5); M: 542 (55.5)	F < 75 years (42.5) vs. (20.3) M, *p* = 0.005		b,c	12		Mortality and hospitalization: Adj. OR for female gender:1.1 (0.65–1.86) Prognosis women < 75 years of age: 7.17 OR (1.79–28.66)	Females, less than 75 years of age, prognosis
Howie-Esquivel (2007) [42]	72; M: 47	62 ± 18					34 were rehospitalized for cardiac reasons within 3 months. Women had a 2.5 times greater risk for rehospitalization than men. 52 days; 40 days non-Caucasian females; > 90 days in males	Women had a 2.5 times greater risk for rehospitalization than men. Non white ethnicity and female gender incurred ≥2 times greater risk of cardiac rehospitalization
Jimenez-Navarro (2010) [38]	Females, 1368 (29) Males, 3351 (71)	Females 64 ± 12, p < 0.001 Males 70 ± 12		a, b, c	40 ± 12, 60% not require readmission	77% not require readmission	Cardiovascular event-free survival: F, 45%, M, 62%	Females higher in heart failure readmission and lower cardiovascular event-free survival, P < 0.001
Macdonald (2008) [25]	With Diabetes, 15,161 Females, 7805 Males, 7356	Females With Diabetes 73.8 ± 10.0 Males with Diabetes 70.0 ± 10.3		a, b, c	*1-month, Diabetic Crude Rate* 7.1 (6.5–7.7) *1-month, Non Diabetic Crude Rate* 5.2 (5.0–5.4)	8.4 (7.7–9.1) 6.8 (6.5–7.0)	Women younger than 65 at both 1 and 5 years have a greater risk for heart failure readmission or death associated with diabetes than in men younger than 65 years and women older than 75 years.	Females < 75 years of age with diabetes
	Without Diabetes, 101,395 Females, 53,578 Males, 47,817	*Females without Diabetes* 77.3 ± 11.5 Males without Diabetes 71.8 ± 12.4			*12-month, Diabetic Crude Rate* 38.0 (36.7–39.3)	38.9 (37.5–40.2)		
					*12-month, Non Diabetic Crude Rate* 29.1 (28.6–29.6)	31.2 (30.7–31.7)		
					*60-Month, Diabetic Crude Rate* 69.8 (68.3–71.3)	70.2 (68.7–71.7)		
					*60-Month, Non Diabetic Crude Rate* 57.6 (57.0–58.2)	58.8 (58.2–59.4)		
Vader (2016) [46]	F: 185 (24.9); M:559 (75.1)	69 (60–78)	6	b,c	1,2		0.74 (0.57–0.98)	Male is lower in readmission or death

^1^a Time to event curves; b - Baseline and Procedural characteristics; c - All relevant outcomes (crude numbers, events, patients, hazard ratios (HR) with 95% CI); Confidence Intervals: 95%; *Adj*. Adjusted, *LVD* Left ventricular dysfunction, *PEF* Preserved ejection fraction, *HF* Heart failure, *M* males, *F* Females. Ratios expressed males to females unless specified

Twelve studies reported higher readmission or composite outcomes in men. Among these, 6 studies demonstrated higher all-cause readmission rates [27, 29, 30, 40, 53, 58] (Table 5) and 4 studies reported higher hazard ratios for composite events [28, 31, 39, 50] (Table 6). In a prospective study, Nieminen et al. [53] reported men having higher rates of all-cause readmission at 1 year compared to a 3-month endpoint. In a Canadian retrospective study, Howlett et al. [28] reported that men were more likely to die than women following rehospitalization at 12 months using the composite measure of death and/or hospital readmission. In total, 5 of 12 studies finding higher readmission rates (not combined with other endpoints) in men examined follow-up durations of 12 months or more.

**Table 5:** Objectives, study population, setting, design and endpoints as reported in studies demonstrating a higher risk for readmission in men. Studies are listed by primary author in alphabetical order

Primary Author (year)	Objectives	Study Population (interventions)	Study Setting (geographical location, recruitment period)	Study Design	Study Endpoints
Alla (2007) [49]	To investigate the association of sex with the risk of adverse events, especially hospitalization for heart failure. To evaluate the association between sex and the risk of mortality and hospitalization, not only for worsening heart failure but other causes, across the clinical syndrome of heart failure.	Patients with clinical heart failure	302 clinical centers (United States and Canada, February 1991 to September 1993)	Retrospective design	All-cause mortality and hospitalization for worsening heart failure, and secondary end points included all-cause hospitalization and cardiovascular hospitalization.
Blackledge (2003) [31]	To compare patterns of admission to hospital and prognosis in white and South Asian patients newly admitted with heart failure, and to evaluate the effect of personal characteristics and comorbidity on outcome	Patients newly admitted with heart failure	UK district health authority (April 1998 to March 2001)	Historical cohort study	Death from any cause (all cause survival) and all cause survival or emergency readmission for a cardiovascular event (event free survival)
Goncalves (2008) [39]	To determine the prognostic value of left ventricular systolic function and identify prognostic indices in patients hospitalized due to HF with preserved and depressed LVSF	Admitted due to decompensated HF	18 months between October 2002 and April 2004, admitted to the Internal Medicine Department	Retrospective design	Primary endpoint was all-cause death or readmission within 6 months
Howlett (2009) [28]	To determine the effectiveness of HF clinics in reducing death or all-cause rehospitalization in a real-world population	Patients with a diagnosis of heart failure	4 heart failure clinics (Nova Scotia, Canada, October 1997 to July 2000)	Retrospective	The primary end point – combined all-cause mortality and hospitalization at the one-year follow-up. Secondary outcomes included the one year total mortality and all-cause hospital readmission rate.
Islam (2013) [58]	Examine demographic and clinical characteristics of patients with CHF who are 65 years of age or older and are and are not readmitted to hospital within 28 days of discharge from an index admission	Older patients with CHF	A large metropolitan public health service (Melbourne, Australia. June 2006 to June 2011)	Retrospective Comparative cohort	Hospital readmission within 28 days
Ieva (2015) [50]	To demonstrate a flexible approach that is able to capture important features of disease progression, such as multiple ordered events and the competing risks of death and hospitalization	Patients with heart failure	Administrative database (Italy, 2000–2010)	Retrospective design	Hospital admissions and death
Madigan (2012) [29]	To determine patient, home health care agency, and geographic (i.e., area variation) factors related to 30-day rehospitalization in a national population of home health care patients with heart failure, and to describe the extent to which rehospitalizations were potentially avoidable	Home health care patients with heart failure	All home care whose care was paid for by the traditional Medicare fee-for-service program (USA, 2005)	Retrospective design	30-day rehospitalization rate
Nieminen (2008) [53]	To evaluate the gender differences in patients hospitalized for acute heart failure in the EuroHeart Failure Survey II	Patents with dyspnoea and verified heart failure	133 Hospitals: university hospitals 47, 49% community or district hospitals, 4% private clinics (30 European countries, October 21st 2004 to August 31st 2005)	Prospective	Gender differences in prescription of HF medication; rehospitalizations and one-year mortality
Ogah (2014) [40]	Examine the rate and predictors of hospital readmission in patients discharged after an episode of heart failure	Patients with heart failure	Private / public primary and secondary health care facilities (Abeokuta, Nigeria, January 2009 to December 2010)	Prospective Study	Hospital readmission
Omersa (2016) [27]	To analyze the readmissions during or following the first HF hospitalization in patients aged 65 years or over, and to evaluate the prevalence of comorbidities and their prognostic implications in terms of mortality and readmission.	Patients aged 65 years or over who had first heart failure hospitalization	Hospitals (Slovenia, 2008–2012)	Retrospective Observational	All cause mortality and readmission within 30 days, and 1 year after discharge from first HF hospitalization
Robertson (2012) [30]	To assess the typical profile, trajectory and resource use of a cohort of Australian patients with heart failure using linked population based, patient-level data	Residents aged ≥45 years with a first (index) admission for heart failure	Admitted Patient Data Collection (New South Wales, Australia, July 2000 to June 2007)	Retrospective Cohort Study Registry	Hospital readmission
Sato (2015) [43]	To compare prognostic risk factors between older and younger chronic heart failure patients	Patients admitted for treatment of worsening CHF	Patients admitted to Fukushima Medical University Hospital, July 2006 and May 2012	Prospective	Cardiac death (death as a result of heart failure and sudden cardiac death) or re-hospitalization as a result of worsening heart failure

**Table 6:** Studies reporting males with significantly higher in all cause re-admissions, death and/or hospital readmission ratios. Heart failure readmission, cardiovascular readmission and mortality/even free survival hazard ratios are also reported. Studies reported by primary author and year of publication and sorted alphabetically

Primary Author (year)	Sample Size n (%)	Mean Age (years)	Type of Reporting by Sex^**1**^	Time to Event (months)	Heart Failure Readmission Hazard Ratio	Death and/or Hospital Readmission Ratio	Other	Significance
Alla (2007) [49]	F:1517 LVD, 407 PEF M: 5273 LVD, 581 PEF	F: 65 ± 12LVD, 69 ± 11PEF; M: 63 ± 11 LVD, 66 ± 9.7	a,b,c	35	Adjusted men vs. women 1.17 (1.06–1.29)		Mortality: Adjusted men vs. women 1.47 (1.33–1.63) All-Cause: Adjusted men vs. women 1.18 (1.11–1.27); Cardiovascular Readmission: Adjusted men vs. women 1.12 (1.04–1.21); When ejection fraction was reduced, 1.19 HR (1.07–1.33) but not preserved HR 0.90: 0.67–1.22	Males, All Cause and lower survival
Blackledge (2003) [31]	F:2913 (50); M:2876 (50)	41–107	b, c	n.r		0.92 (0.85–0.98)	Mortality: 0.88 (0.82–0.96)	Males, Death and/or Hospital readmission and lower survival
Goncalves (2008) [39]	F(54.3); preserved LVSF F(72.9),M(26 (27.1); depressed LVSF F (45), M (113, 54.3)	72.7 (1.6); preserved LVSF 73.3 ± 11.2 vs. depressed LVSF 70.7 ± 12.7, *p* = 0.13	b, c	6		M preserved LVSF 2.04 (1.08–3.84); M Depressed LVSF 0.64 (0.42–0.96)		Males with preserved and depressed LVSF, Death and/or hospital readmission
Howlett (2009) [28]	F:364(37), M:620 (63)	68 ± 13	b,c	12		1.21 (1.06–1.37)		Males, death and/or hospital readmission
Islam (2013) [58]	F:313 (49.7), M:317 (50.3)	65–74:22.4 75–84:47.6 85+:30.0	b, c	n.r.			All Cause Readmission: 1.22 (1.03–1.46)	Males, All Cause
Ieva (2015) [50]	F:8114 (53.04), M:7184 (46.96)	F:79.6(11.4), M:71.5 (12.88)	a, b	n.r.			n.r.	Males
Madigan (2012) [29]	F:45429 (61)		c	1			All Cause Readmission: 1.079 (1.047, 1.112)	Males, All Cause
Nieminen (2008) [53]	3580, F: 1384 (29); M:2916(61)	F: 73.1 ± 12.0, p < 0.001; M: 67.8 ± 12.4	a,b,c	3,12			Mortality: 1.04 (0.79–1.37); All-Cause Readmission: Age-adjusted, 0.84 (0.74–0.96); Event-free survival: Death, myocardial infarction or stroke, 10.1: 9.7; 0.95 (0.76–1.20)	Males, All Cause
Ogah (2014) [40]	F: 124(47), M 138 (53); rehospitalized m 21 (65.6); not hospitalized m 117 (50.9)	Readmitted 61.7 ± 14.0 vs non readmitted 56.1 ± 15.4, *p* = 0.026	b, c	1, 6			All-Cause Readmission: F: 11(8.9%); M: 21(15.2%); OR 0.54 (0.25–1.18); Adjusted for women 0.33(0.14–0.79)	Males, All Cause
Omersa (2016) [27]	F: 21711 (59); M: 15113(41)	F: 65–74:19%, 75–84:48%, 85+:33%; M:65–74:36,75–84:48%;85+:16%	c	n.r.			Mortality: 65–74: 0.808 (0.745–0.875) 75–84: 0.848 (0.807–0.891) 85+: 0.840 (0.785–0.898); All Cause Readmission: 65–74:0.872 (0.814–0.934) 75–84:0.869 (0.825–0.915) 85+:0.855 (0.784–0.931)	Males, and lower survival
Robertson (2012) [30]	F:14557 (50); M: 14604 (50)		b,c	n.r.			All-Cause Readmission: 0.93 (0.89–0.96)	Males, All Cause
Sato (2015) [43]	F: 122; M: 476 (79.6)		b,c	26			Cardiovascular Readmission: Multivariable male 1.851 HR (1.237–2.771)	Males, Cardiovascular

*F* Female, *M* Male; Male to Female ratio assumed unless specified. ^1^ a- Time to event curves; b - Baseline and Procedural characteristics; c - All relevant outcomes (crude numbers, events, patients, hazard ratios with 95% CI); *LVD* Left Ventricular Dysfunction, *PEF* Preserved Ejection Fraction, *LVSF* Left Ventricular Systolic Function, *n.r.* Not reported

Eighteen studies reported no sex differences in readmission rates between men and women (Table 7).

**Table 7:** Objectives, study population, setting, type of study, and study endpoints as reported in studies with similar rates between males and females. Studies reported by primary author in alphabetical order

Primary Author (year)	Objectives	Setting (geographical location, recruitment period)	Study Design	Study Endpoints
Ahmed (2014) [57]	Examined the impact of gender on a wide variety of major natural history endpoints in a propensity matched population of ambulatory chronic HF patients in which men and women were well balanced on all measured baseline covariates	302 clinical centers across the United States (186 centers) and Canada (116 centers) between January 1991 and August 1993.	Retrospective observational	Mortality, hospitalizations (all cause, cardiovascular causes and HF)
Bradford (2016) [32]	To evaluate the diagnosis and timing and to identify patient and clinical characteristics associated with 30 day readmissions among heart failure patients.	Acute care hospitals (San Diego, US, October 2009 to November, 2014)	Retrospective	30-day Readmissions
Chang (2014) [48]	To study sex differences in clinical characteristics and outcomes among multi-ethnic Southeast Asian patients with hospitalized heart failure	Hospitals in the Southeast Asian nation of Singapore, January 1, 2008 to December 31, 2009	Prospective	Length of stay, in hospital mortality and rehospitalisation
Chun (2012) [35]	Examined a patient cohort discharged after being newly hospitalized for HF and followed them over their lifetime for all cardiac and noncardiac hospitalizations that occurred until death. Examined patterns of hospitalization and recurrent cardiovascular events and the association of sex, presence of HFrEF versus HFpEF, and ischemic versus nonischemic etiology on hospitalizations	Hospitals (Ontario, Canada, April 1999 to March 2001)	Retrospective	Recurrent hospitalizations, cardiovascular events, and survival
Eastwood (2014) [33]	To identify factors associated with risk of all-cause and HF-specific readmissions within 7 and 30 days of discharge	Acute care hospital in Alberta from April 1, 2002 to March 31, 2012	Retrospective	7-and 30-day readmission for all causes, 7-and 30-day readmission for HF
Gevaert (2014) [47]	To compare the incidence and treatment of atrial fibrillation on admission between men and women admitted with acute heart failure	2 Belgian hospitals, Nov 2006 to May 2012; Patients included in the prospective BIO-HF registry (evaluates all patients admitted with the New York Heart Association class 3–4)	Prospective design	One-year all-cause mortality or readmission for HF. Secondary endpoints were in-hospital mortality and restoration of sinus rhythm at discharge
Jenghua (2011) [36]	To determine early readmission rate after discharge among patients with principal diagnosis of CHF and (2) identify predictors of readmission within 30 days after discharge for this group of patients	Tertiary care hospital in a large metropolitan area of Phitsanulok Province, Thailand	Retrospective	Rate of readmission after discharge; predictors of readmission
Lee (2004) [37]	To evaluate the effect of gender on the risk of all-cause rehospitalization and that specific to heart failure in a diverse contemporary cohort of adults who had been hospitalized with HF	16 Kaiser Permanente of Northern California facilities (July 1, 1999 to June 302,000)	Retrospective cohort	Any rehospitalisation and readmission due specifically to heart failure
Mullens (2008) [54]	To investigate whether there is gender-specific differences in clinical presentation, response to intensive medical therapy, and outcomes in patients admitted with advanced decompensated heart failure.	Dedicated heart failure intensive care unit in clinic (USA, 2000 to 2006)	Retrospective	All-cause mortality, all-cause mortality or cardiac transplantation and first readmission for heart failure after discharge
Nieminen (2008) [53]	To evaluate the gender differences in patients hospitalized for acute heart failure in the EuroHeart Failure Survey II	133 Hospitals: university hospitals 47, 49% community or district hospitals, 4% private clinics (30 European countries, October 21st 2004 to August 31st 2005)	Prospective	Gender differences in prescription of HF medication; rehospitalizations and one-year mortality
Ogah (2015) [44]	To evaluate the sex differences in acute heart failure in sub-Saharan Africa	12 Cardiology units (9 sub-Saharan African countries: Cameroon, Ethiopia, Kenya, Mozambique, Nigeria, Senegal, South Africa, Sudan and Uganda, July 12,007 to June 302,010)	Prospective	Length of hospital stay, mortality rates, and all-cause re-admission
Opasich (2004) [41]	To identify differences between sexes in the clinical profile, use of resources, management and outcome in a large population of ‘real world’ patients with heart failure	167 Cardiology (CARD) and 250 internal medicine (MED) departments (Italy, February 14, 2000 to February 25, 2000)	Retrospective	Number of cardiovascular procedures and diagnostic, and pharmacological therapy, in-hospital mortality
Otero-Ravina (2009) [56]	Characterization of current morbidity and mortality among heart failure in Galicia together with their main determinants	Eight geographical areas of Galicia, year 2006	Prospective	Survival rates
Sajeev (2017) [45]	Study the demographical and clinico-pathological characteristics of patients presenting with heart failure and evaluate the 1 year outcomes and to identify risk predictors if any	A tertiary care centre (South India, April 2013–September 2014)	Prospective	Mortality and/or re-hospitalization due to HF
Schwarz (2003) [55]	To evaluate whether severity of cardiac illness, cognitive functioning, and functional health of older adults with heart failure (HF) and psychosocial factors related to caregiving are predictive of hospital readmissions for those with HF	2 Community hospitals (Ohio, US)	Prospective	3-month re-admission
Sheppard (2005) [34]	To explore gender differences in therapy, resource utilization, and clinical outcomes in patients who had CHF	Quebec hospital summary database linked to provincial physician and drug claims databases, January 1998 and December 2002	Retrospective	Procedure, medical therapy and re-hospitalizations, emergency room visits
Tarantini (2002) [51]	Evaluate the clinical characteristics, 1-year prognosis and therapeutic approach of heart failure with a preserved left ventricular systolic function in a large multicenter registry of patients referred to specialized heart failure clinics	133 Centers of the ANMCO working group on heart failure, March1995 to January 1999	Prospective	Use of cardiovascular medications, hospitalizations (all-cause for cardiovascular events and for worsening CHF)
Zsilinskza (2016) [52]	Evaluate sex differences in patients with HFpEF that presented to the ED with acute HF, regarding presentation, treatments, and outcomes.	83 Hospitals (United States, January 2004 to September 2005)	Retrospective	Emergency department therapies and management, hospital length of stay, in-hospital mortality, post-discharge outcomes

*ANMCO* Associazione Nazionale Medici Cardiologi Ospedalieri (National Association of Hospital Doctors Cardiologists), *N.R* None reported, *HFpEF* Heart failure preserved ejection faction, *ED* Emergency department, *EF* Ejection Faction, *AHF* Acute heart failure, *HF* Heart failure, *CHF* Congestive heart failure, *ICD* International Statistical Classification of Diseases and related health problems

### Non-HF cardiovascular causes of readmission

The observed sex differences in readmission rates could be potentially explained by several factors including patient characteristics and differences in processes of care. Vader [46] reported risk factors for post-discharge readmission or death in patients treated for acute heart failure, including male sex, non-use of angiotensin-converting enzyme inhibitors (ACEI) or angiotensin receptor blockers (ARB), lower baseline sodium, non-white race, lower systolic blood pressure at discharge or day 7, increased length of stay, and depression. In addition, both Vader [46] and Howie-Esquivel [42] reported a two-fold increase in cardiac readmission rates for women whose ethnicity was Caucasian. Older age and the comorbidities that are associated with aging were reported to be risk factors for readmission [27, 30]. Comorbidities including myocardial infarction, chronic kidney disease, cancer, and diabetes mellitus were cited as predictors of increased readmission risk [27].

Some co-variates such as diabetes, valvular heart disease, blood pressure, and atrial fibrillation were sex-sensitive, and could potentially have a role in differential risk of readmissions in women and men. Diabetes and anemia were significantly more frequent in women [53]. Women more often presented with atrial arrhythmia and atrial fibrillation, and men with ventricular arrhythmias [53]. Women were more likely to present with valvular heart disease, hypertension, and preserved left ventricular function, but also less likely to be diabetic or smokers [52, 53]. The presence of coronary disease and an ischemic etiology may modify HF outcomes in women and men. Specifically, Mullens [54] reported better survival rates for women with a nonischemic cause, whereas there was a trend toward worse survival in those with ischemic heart failure.

The effects of ejection fraction on sex-specific readmission rates are variable. Alla [49] reported that males had a relative risk of hospitalization for heart failure that was greater than females when the ejection fraction was reduced but not when preserved. The authors’ survival analysis showed an advantage for females with HF and reduced ejection fraction [49]. In a retrospective study, Goncalves et al. [39] found that males with preserved and reduced left ventricular systolic function were more likely to die and/or be re-admitted within 6 months.

Howlett [28] found that women were more likely to receive usual care rather than specialized heart management from a clinic (52% vs. 37%). Nieminen [53] observed higher rates of readmission in men even when both sexes were treated as frequently and as long in the different types of wards (internal medicine, cardiology, intensive care unit and cardiac care unit). The initial place of presentation for both women and men was the emergency department followed by admission to the general ward [36].

There was some indication that whether women were alone, supported socially by a partner, or working could impact readmission rates. Niemminen [39] discussed that readmitted women were more often retired, living alone or in special accommodation.

### Differences in interventions

Nieminen [39] described that women underwent significantly fewer invasive procedures. However, Chang [36] found no sex differences in the use of critical life-saving procedures such as renal replacement therapy, mechanical ventilation, defibrillation or cardiopulmonary resuscitation. Zsilinszka [44] also did not note any sex differences in use of dialysis or mechanical ventilation. It appeared that where differences in procedures were observed, there was some clinical discretion involved and they were not necessarily immediately critical for sustaining life. For example, invasive procedures such as coronary angiography were performed less often in women than men (1.4% vs. 2.8%, *p* < 0.001) [36]. Sheppard [32] also found that women underwent fewer non-invasive assessments of ventricular function and ischemia and had fewer revascularization procedures.

More studies, in general, reported that women were less likely to receive evidence-based therapies than men. Men were more often prescribed β-blockers, vasodilators, and antiplatelet agents than women and were administered higher mean doses than women [38]. Women were also less frequently prescribed ACE inhibitors [34]. Chang [48] noted that women were less likely to receive evidence-based therapies upon discharge compared to men. In contrast to the above, Zsilinskza [52] did not note any sex differences in HF therapies, such as administration of diuretics or vasodilators. A potential reason for the similar rates of some drug classes in women is that the higher prevalence of hypertension in women may have overlapping indications for HF. Therefore, increased beta-blocker therapy in women, for example, may reflect management of hypertension and HF with preserved ejection fraction [34].

## Discussion

Findings from this scoping review confirm that while most of the studies that included sex-based analyses showed no differences in readmission rates following HF hospitalization, several factors still point to the need of targeted sex-based management for at-risk populations. The rise in hospital readmissions is a global concern and is often used as a quality benchmark for health care systems. Hospital readmission is a considerable burden on the individual from a cost perspective and the related treatment costs and hospital resources tax the health system. This review is the first to use a systematic method to describe the literature for assessing readmission rate differences in men and women with an index HF hospitalization.

We have observed that there might be an interplay between sex, timing post-discharge, and readmission rates. Studies in which women were more likely to have higher readmission rates than men tended to have durations of follow-up of less than 12 months. This finding suggests that short-term follow-up, improved self-management and early care following discharge from the hospital may be needed. Reducing readmission rates in women may necessitate consideration of sex-specific roles and supports for women who live alone. In contrast, readmission rates appeared to be higher for men in studies with longer durations of follow-up (> 12 months) [28, 53].

Few studies with a sex and gender sensitive approach have been conducted prospectively. HF readmission is more typical of patients who are men with reduced ejection fraction than women [59]. Two of the retrospective studies which contained information on LVEF [39, 49] indicated that the tendency for readmission was higher in men as compared to women. When the totality of studies was examined, including those with or without LVEF assessment, more studies indicated a higher readmission rate for men overall.

Sex and gender differences may relate in part to compliance with pharmacological treatments. Analysis of the EuroHeart Failure Survey indicated that compliance with pharmacological therapies differed between men and women. Men may be less compliant to pharmacological treatments, following discharge after an index diagnosis of heart failure. As future next steps, education with good integrated care and follow up conversations with practitioners warrants investigation leading to improved health outcomes and reduced readmission rates among men. The greater use of invasive diagnostic procedures in men may also relate to the observed early differences in outcomes since these procedures may impact early outcomes reflecting the care received during the acute care hospitalization.

A sex specific approach to post-discharge heart failure care may require better access points of care for certain populations. For example, earlier readmission risk in women may suggest that improved post-discharge transitional care, ongoing physician follow-up in the near-term, home visitation by nurses, and remote patient monitoring could be particularly useful. In men, later readmissions may indicate the need for long-term follow-up, ensurance of medication compliance, and early treatment of conditions such as ischemic HF, which can lead to late readmissions downstream. Further, future studies should report sex-specific readmission and mortality outcomes separately, since the composite outcome of death or readmission may mask informative underlying patterns. Sex-based approaches to pharmacotherapy also warrant greater exploration. For example, it is known that absorption rates and metabolism of digoxin are different between men and women [14]. Thus, serum digoxin should be administered in lower doses to women to avoid toxicity because of these pharmacokinetic differences [60].

### Strengths and limitations

Given the large sample of papers in our database, we focused on sex-sensitive factors which could be part of a global strategy to reduce readmission rates. Our methodology was robust as we included a consultation panel of experts, which is an optional step in the Arksey and O’Malley framework. We also consulted individually with a professional librarian, a cardiologist, and sex and gender experts. We linked the references of relevant studies when a primary research study cited either appendices, supplementary material, or pilot studies or if the same author wrote a dissertation and an abstract in conjunction with a journal article [42, 61, 62].

There are some limitations to our scoping review. A primary objective needed to state evaluations of sex and gender for a study to be included in this scoping review. Secondary or subgroup analyses may not result in conclusive findings without a power calculation for factors related to sex. Further, without a pre-specified sample size that powers these secondary analyses, even if findings are positive, may not be clinically relevant. We also found that sex and gender terminology was often confused which could conflate study findings and may not clearly demonstrate how the interrelated nature of these concepts may impact men and women. The time spent in observation units by hospitals and the use of emergency department might be explored in future studies. This scoping review, while providing a description of the research on readmission rates as it relates to sex and gender to date, is limited in its ability to demonstrate trends over time. Finally, the observations of the potential interaction between sex-specific readmission risk and time horizon can only be considered hypothesis generating in this review, and indeed many studies showed no difference. This interaction between sex and time horizon may need to be confirmed in different jurisdictions, accounting for the competing risks of death, before implementing policies directed toward reducing early vs. later-term readmissions.

## Conclusion

Overall, there is an increase in reporting of sex and gender differentiated data; however, we found that in most studies, this was not explicitly stated in the primary objectives. We found more papers reported that men with heart failure had significantly higher readmission rates compared to women with heart failure. The effect of sex on readmission may have been dependent on follow-up duration, with longer follow-up duration favoring higher readmission rates among men. Readmission reduction programs could include targeted educational approaches and conversations with practitioners following discharge about medication compliance and management strategies. In addition, we encourage the use of singular rather than composite measures (i.e. combination of mortality and readmission), the latter having the potential to mask important sex-specific associations. Future studies would be needed to investigate heart failure in relation to readmission rates to examine sex and gender differences and their effects over time.

## Data Availability

See supporting data. All other data are systematically-reviewed from previously published material, and therefore available in the public domain.

## Appendix

Peer Review Assessment

TRANSLATION A. No Revisions

BOOLEAN AND PROXIMITY OPERATORS A. No revisions

SUBJECT HEADINGS b. Revision(s) suggested

There is one missing MeSH. Line #4 should contain exp. patient readmission. This, however, would be picked up in the PubMed search.

TEXT WORD SEARCHING.A. No revisions

SPELLING, SYNTAX, AND LINE NUMBERS A. No revisions

LIMITS AND FILTERS A. No revisions

OVERALL EVALUATION A. No revisions

Database: Ovid MEDLINE(R) In-Process & Other Non-Indexed Citations and Ovid MEDLINE(R) < 1946 to Present>
